# InChI: a user’s perspective

**DOI:** 10.1186/1758-2946-4-34

**Published:** 2012-12-13

**Authors:** Steven M Bachrach

**Affiliations:** 1 Department of Chemistry, Trinity University, 1 Trinity Place, San Antonio, TX 78212, USA

## Abstract

Exchange of chemical structures between practicing chemists is essential to chemical communication. The International Chemical Identifier (InChI) provides a means for lossless communication of structures without resort to any proprietary software or databases nor does it require any payment or royalty fees. This perspective describes why the InChI is valuable to all chemists and how it will be an essential component of creating the chemical web.

## Commentary

**Figure 1:**
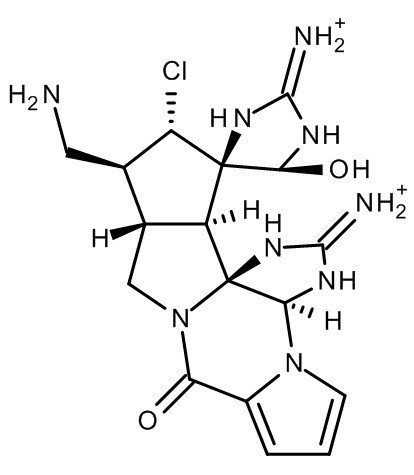
Structure of palau'amine.

Suppose you are working on an exciting project and you wish to tell a friend about the molecule you’ve been investigating. As a chemist, the best way to convey the molecule to your friend is with a drawing, such as Figure 1. If, however, you are speaking to your friend on the phone, you have no easy means of communicating this image. You might then resort to using its name. The trivial name of the compound in Figure 1 is palau^′^amine (actually the diprotonated form of palau’amine) [1], and unless your friend is a natural products chemist, she is unlikely to be able to extract the structure from that name. The IUPAC nomenclature system is designed for just such a case: a means of unambiguously describing the structure of a compound. So you tell her that the name of the compound you are working on is (3aS,5'S,10aS,11S,12S,13R,13aS,13bR)-2,2'-Diamino-11-(aminomethyl)-12-chloro-5'-hydroxy-8-oxo-1,1',3a,5',10a,11,12,13a-octahydro-8H,10H-spiro[cyclopenta[3,4]pyrrolo[1,2-a]imidazo[4,5-b]pyrrolo[1,2-d]pyrazin-3-ium-13,4'-imidazol[3]ium]. I suspect that neither of you is at all sure of what the structure of Figure 1 really is! You might decide to tell your friend the CAS registry number [2] of the compound, but this entails that both you and your friend have access to the CAS registry. In other words, while the CAS RN would uniquely define the compound, it requires a transactional cost on both the sender’s and receiver’s part in order to process the communication; just to tell your friend the structure both you and she will have to pay to use the proprietary CAS system! Because the registry number is simply a numeric pointer into the database, there is no algorithmic means for turning a CAS RN into a structure [3].

Let’s change the scenario to one where you need to communicate many different structures, and you want to use these structures within a database containing a very large number of compounds. For example, you are registering a set of new compounds from the medicinal chemistry group and you need to insure that these are all new and unique. Having a collection of images is not going to work; it will take too much time to visually compare the images with the ones in the database. You might utilize *SMILES*[4], a linear coding scheme for compounds. Unfortunately, there are different versions of SMILES, often giving very different representations, and the modifying terms “canonical” and “isomeric” are frequently in conflict. SMILES strings cannot be guaranteed without resort to purchased software, and differences in SMILES strings between software versions are known.

What is needed is some means of encoding a chemical structure into a computer-readable string, transmitting this string, and then decoding it back to a chemical structure without any impediments. Communication of a chemical identity should be done without having to incur any fee and should be done without any confusion or ambiguity.

This is exactly the goal of the InChI project: barrierless communication of chemical structures. InChI stands for “International Chemical Identifier”. It provides a string of characters that uniquely encode a chemical structure; for every molecule there is one and only one InChI and each InChI string describes a single compound (though depending on the tautomer treatment setting, a single InChI might represent a family of tautomers) [5].

The key component of InChI-enabled communication is a software package [6] that encodes a chemical structure into a string of letters and numbers called an identifier. This software was developed under the auspices of the International Union of Pure and Applied Chemistry and the National Institute of Standards and Technology. The InChI Trust [7] acts as the oversight body to insure that the software is maintained, and working with IUPAC, insures that the InChI algorithm remains an open standard. The software is Open Source, and thus can be used and repurposed without a fee. In fact, many software companies have incorporated the InChI algorithm within their own chemical drawing package, such as ACD/ChemSketch [8], Accelrys Draw [9] and ChemDraw [10].

The InChI software will also decode an InChI string into a chemical structure. If both sender and receiver have the InChI software, they can communicate chemical structures simply by encoding the structure into the string using the free software tool, transmit the unique identifier, and the receiver then decoding (with the identical software) the string back into a structure in a completely lossless and no-cost transaction.

The InChI string is designed to be flexible and extensible. It is broken into layers that represent different components of chemical structure [5]. So, for example, the InChI string for palau'amine is

InChI=1S/C17H22ClN9O2/c18-10-6(4–19)7-5-27-11(28)8-2-1-3-26(8)12-17(27,25-14(20)22-12)9(7)16(10)13(29)23-15(21)24-16/h1-3,6-7,9-10,12-13,29H,4-5,19H2,(H3,20,22,25)(H3,21,23,24)/p+2/t6-,7-,9+,10+,12+,13+,16+,17-/m1/s1.

The first layer, *1S* in this case, indicates that this is the standard InChI version 1. The second layer, *C17H22ClN9O2*, is the molecular formula. The other layers encode the atomic connectivity and the stereochemistry.

The InChI string can be quite daunting and one might look at the length of it for palau’amine and shake one’s head in disbelief. It is important to remember though that the InChI string is not meant for human use; rather, it is meant for computers to use and process, and computers have no fear of a long character string.

One potential use of the InChI string is to make chemical structures discoverable on the Internet. The length of the InChI string is a problem in searching on the Internet, where many popular search engines limit a search term to a specific fixed length. In order to facilitate discoverability, the InChIKey was developed [11]. The key is a near-unique encoding of a (long) InChI string into a short string of 27 characters. (For example, the InChIKey of palau'amine is VYOQBYCIIJYKJA-VORKOXQSSA-P). This is accomplished by a hashing technology, which does not preserve uniqueness. Furthermore, the InChIKey is not reversible by algorithm. Nonetheless, the InChIKey provides a mechanism for making chemical structures available through the web. For example, on my own blog, *Computational Organic Chemistry Blog*[12], I include InChI and InChIKey of the compounds I discuss. Someone can then submit an InChIKey into their favorite search engine and find my blog posts that discuss that compound.

What can InChI do for you? In an ideal world, one would be using InChI strings extensively without ever even being aware of them. One can think of them as analogous to the IP addresses that identify our computers on a network. Most of the time, we never need to know what IP address has been assigned to our personal computer; just as long as we can connect to the web, we are happy. InChIs can offer us the same working condition. A chemist could use whatever structure drawing tool they wish to draw a compound, and then pipe this into a search engine or into a database or send it off to a friend or embed it into a manuscript, and the InChI comes along behind the scenes, ready for computers to use to link documents together and make these documents chemically discoverable.

As more and more publishers, chemical suppliers, database producers, *chemists* embed InChI into their documents and programs and Internet resources, the more connections (links) will be made based on chemical structures. The InChI will guarantee the authenticity of the link between documents, insuring that the chemical structure requested is the chemical structure being delivered. The semantic web can be made chemically-rich through widespread adoption and use of InChIs.

It is important to recognize that in no way does InChI replace or make outmoded any other chemical identifier. A company that has developed their own registry system or one that uses one of the many other identifiers, like a MOLfile [13], can continue to use their internal system. Adding the InChI to their system provides a means for connecting to external resources in a simple fashion, without exposing any of their own internal technologies.

The InChI project is ongoing; not all of chemistry is yet covered by the software. The vast majority of organic compounds can be encoded into InChIs, but inorganic and organometallic compounds are still works in progress. Encoding a chemical reaction, a reaction InChI [14], is an actively pursued extension. Polymers pose an interesting challenge as does the ability to encode Markush structures. Excited states, transition states, rotoxanes, host-guest complexes and biologics are missing. The InChI Trust has plans to address these, and other, categories in due time.

So, in the end, a request to the reader: to utilize InChIs in your own web communications, to encourage others to do the same, to ask publishers and suppliers to adopt the use of InChI, to help develop the InChI standard by extending it into new chemical domains, to support the InChI trust, to help build the chemical web!

## Competing interests

The author’s wife is employed at Accelrys, Inc.
